# An Automated Tobacco Cessation Intervention for Emergency Department Discharged Patients

**DOI:** 10.5811/westjem.2021.2.49489

**Published:** 2021-07-19

**Authors:** David T. Chiu, Ronald Lavoie, Larry A. Nathanson, Leon D. Sanchez

**Affiliations:** * Beth Israel Deaconess Medical Center, Department of Emergency Medicine, Boston, Massachusetts; † Harvard Medical School, Department of Emergency Medicine, Boston, Massachusetts; ‡ Northern Arizona Healthcare, Department, Flagstaff, Arizona; § Brigham and Women’s Hospital, Department of Emergency Medicine, Boston, Massachusetts

## Abstract

**Introduction:**

Nearly 14% of US adults currently smoke cigarettes. Cigarette smoking causes more than 480,000 deaths each year in the United States. Emergency department (ED) patients are frequently asked for their use of tobacco. Manual selection of pre-formed discharge instructions is the norm for most ED. Providing tobacco cessation discharge instructions to ED patients presents another avenue to combat the tobacco use epidemic we face. The objective of the study is to evaluate the effectiveness of an automated discharge instruction system in increasing the frequency of discharging current tobacco users with instructions for tobacco cessation.

**Methods:**

The study was done at an urban academic tertiary care center. A before and after study was used to test the hypothesis that use of an automated discharged instruction system would increase the frequency that patients who use tobacco were discharged with tobacco cessation instructions. Patients that were admitted, left against medical advice, eloped or left without being seen were excluded. The before phase was from 09/21/14–10/21/14 and the after phase was from the same dates one year later, 09/21/15–10/21/15. This was done to account for confounding by time of year, ED volume and other factors. A Fisher’s Exact Test was calculated to compare these two groups.

**Results:**

Tobacco cessation DC instructions were received 2/486 (0.4%) of tobacco users in the pre-implementation period compared to 357/371 (96%) in the post-implementation period (p < 0.05).

**Conclusions:**

The automated discharge instructions system increases the proportion of tobacco users who receive cessation instructions. Given the public health ramifications of tobacco use, this could prove to be a significant piece in decreasing tobacco use in patients who go to the emergency department.

## INTRODUCTION

Nearly 14 of every 100 U.S. adults aged 18 years or older (14.0%) currently smoke cigarettes. Cigarette smoking causes more than 480,000 deaths each year in the United States.^1^,^2^ Patients are usually asked about tobacco use by nursing in the emergency department (ED) as part of a set of standardized questions during the initial triage process^3^ However, this information is rarely addressed by the physician unless the tobacco use is relevant to the presenting complaint, such as with acute respiratory illness.^4^,^5^ Patients frequently leave the ED, with the emergency physician aware of their patient’s tobacco use however with only a small minority stating that they provided any intervention to help the patients quite tobacco use.^4^ ED patients often times are interested in quitting smoking however lack the resources to do it.^6^ Prior studies have shown only 27% of emergency physicians routinely asked patients to quit smoking.^7^

Perceived barriers by physicians to addressing tobacco use in the ED include lack of training, resources, and time as ED volumes continues to climb.^7^,^8^ This presents a challenge for emergency physicians as the high volume of patients make it challenging to address non-emergent issues such as tobacco use. However, with electronic health records (EHR) becoming ubiquitous, studies have shown improvement of smoking cessation practices through automated reminders.^9^ Printed self-help materials help more people to stop smoking than no intervention.^10^ Therefore, providing tobacco cessation discharge instructions to ED patients presents another avenue to combat the tobacco use epidemic we face.

The aim of our study is to evaluate the effectiveness of an automated discharge instruction system in increasing the frequency of discharging current ED tobacco users with instructions for tobacco cessation.

## MATERIALS AND METHODS

### Study Design and Setting

The study was granted Institutional Review Board exemption status. The setting of the study is an urban academic tertiary care center with an affiliated three-year emergency medicine residency. The hospital uses a homegrown EHR and does not use a proprietary vendor. The automated discharge instructions system was specifically designed for this EHR, which the hospital continues to use at the time of publication.

A before and after study was used to test the hypothesis that use of an automated discharge instruction system (which automatically detects for tobacco use) would increase the frequency of tobacco cessation discharge instructions usage. All patients who were discharged from the ED during the study period were enrolled. The hospital does not routinely see patients under the age of 18, however any pediatric patients that were seen and discharged in the ED were included in the study. Patients that were not properly discharged were excluded including admitted, left against medical advice, eloped, expired, transferred, or left without being seen. Sample size calculations were performed with an alpha of 0.05 and a power of .80 to detect a 5% increase in the inclusion of tobacco cessation discharge instructions in the post implementation group. A convenience sample of patients was collected during two, 31-day time periods. The intervention was deployed on November 20, 2014. The before group data were collected from September 21, 2014 to October 21, 2014, thirty days prior to intervention. Patients from the exact time frame one year later comprised the after group, September 21, 2015 to October 21, 2015.

### Methods of Measurement

As part of the normal triage screening process, patients are asked a brief social history by the triage nurse, including the use of tobacco, alcohol or other illicit drugs. For tobacco use specifically, patients are specifically asked by the triage nurse, “Do you currently use tobacco products?” The responses are captured in dichotomous structured data elements with the option of further detail in free text comment box. This did not change pre- or post-intervention. These patients in their discharge instructions had a standard discharge instructions attachment automatically included in their discharge paperwork. Patients who had tobacco screening questions asked after discharge paperwork initiated would not have the tobacco cessation instructions automatically included. Patients were considered to be tobacco users if on review of their completed chart, the patient responded affirmatively to either anyone on the care team (e.g., nursing, attending physician, resident physician, supervising senior resident) asking them about tobacco use.

### Outcome Measures

The primary outcome measure in this study is the inclusion of tobacco cessation discharge instructions in patients discharged from the ED.

### Primary Data Analysis

In order to ensure that the before and after study populations did not statistically demonstrate any differences, baseline characteristics between the two groups were tested for significant different. Age collected in years was treated as a normal distribution and a two-sample t-test with unequal variances was used. Gender (male/female), race (white/non-white), language (English/non-English) and tobacco (use or no use) were dichotomous variables and a Fisher’s exact test was used. Emergency Severity Index (ESI) is an ordinal variable and a Pearson’s chi-squared test was used. Length of stay (in minutes) was found to be not normally distributed and a Wilcoxon rank-sum test was used. Lastly, inclusion of the standard tobacco cessation instructions into the discharge paperwork was dichotomous so Fisher’s exact test was used.

## RESULTS

### Characteristics of Study Subjects

A total of 2824 patients were discharged from the ED during the before phase compared to 2818 in the after phase. The before and after group did not demonstrate any significant differences in various characteristics based on testing. Table 1 shows these attributes in the before and after populations as well as the statistical testing results.

### Main Results

Tobacco cessation discharge instructions were received in 2 out of the 486 (0.4%) of tobacco users in the pre-implementation period compared to 357 out of the 371 (96%) in the post-implementation period. The Fisher’s Exact test was significant with a p-value of <0.001.

## DISCUSSION

The automated discharge instruction system significantly increased the number of tobacco-using patients who were subsequently discharged with tobacco cessation counseling instructions. Given the public health ramifications of tobacco use, this could prove to be a significant piece in decreasing tobacco use in tobacco using patients who are discharged from the ED.

Prior to implementation of the automated process, providers manually selected tobacco cessation discharge instructions in appropriate situations in less that 1% of patient encounters, a similar rate to prior studies.^3^ With a simple automated intervention this rate increased to over 95% adherence, thereby circumventing the prior barriers emergency providers encountered. Fourteen patients in the after group who did use tobacco did not end up getting the cessation instructions because they not properly triaged due to. acuity thereby bypassing triage, language barrier, and nursing oversight. Tobacco users were based off of chart review of a combination of triage, physician and nursing documentation so additional patients were captured that use tobacco that were not initially picked up at triage.

Tobacco cessation is merely one of many non-emergent health care issues that emergency physicians encounter on a daily basis with greater adoption of EHRs, there is ample opportunity to easily identify non-emergent but important patient issues (such as hypertension, hyperglycemia, alcohol abuse) and automate a structured response in an effort to deliver better care.

## LIMITATIONS

One limitation of the study is that this is a before-after study and therefore subject to potential confounders. Thus, the study periods were chosen exactly 1 year apart as this would try to account for some confounding. Some of the more common populations characteristics were compared in our study to ensure two similar study populations. No significant operational or staffing changes were made between the two study periods.

Other limitations include whether the outcome measure of increasing the rate of tobacco users presenting to the ED who are subsequently discharged with tobacco cessation instructions is clinically relevant. By automating detection and inclusion of discharge materials for patient education, this offloads this simple but easily forgotten task to the EHR thereby vastly increasing the likelihood of attaching instructions to help the patient cease tobacco use. The use of a custom homegrown EHR is another limitation, as most hospitals have shifted to a commercial vendor. However, the concept and programming implementation for this intervention should be easily reproducible with minimal cost and effort.

Future studies include long term follow up of those who received tobacco cessation discharge instructions compared to those that did not and observing for decrease in tobacco use. Other areas of research include using similar automated discharge instructions for other common, overlooked chronic conditions in ED discharge patients such as hypertension and blood sugar management.

## CONCLUSIONS

Using an automated discharge instruction system can help emergency physicians increase the frequency of providing written instructions on tobacco cessation to users, which has previously been shown to help more people to stop smoking than no intervention. Tools such as automated discharge instructions provide a means of addressing incidental, chronic issues that busy emergency physicians might otherwise overlook.

## Figures and Tables

**Table 1:** Characteristic comparison between the before and after implementation of the automated discharge instructions module.

	Before group	After group	P-value
Age (years)	47.3	46.9	0.53
Gender (% female)	59.8	56.8	0.94
ESI (1–5)	2.86	2.82	0.89
Race (% white)	54.8	52.9	0.79
Language (% English)	90.4	89.4	0.47
LOS (minutes)	299	320	0.58
Tobacco (% use)	17.2	13.2	0.24

*LOS*, length of stay; *ESI*, emergency severity index.
